# Infectious disease hotlines to provide advice to general practitioners: a prospective study

**DOI:** 10.1186/s12913-023-09515-3

**Published:** 2023-05-17

**Authors:** Anna Luce Sette, Patrice François, Philippe Lesprit, Virginie Vitrat, Olivier Rogeaux, Emma Breugnon, Marion Baldeyrou, Véronique Mondain, Bertrand Issartel, Solen Kerneis, Sylvain Diamantis, Delphine Poitrenaud, Bastien Boussat, Patricia Pavese

**Affiliations:** 1 Médecine Générale, Centre Hospitalier Universitaire Grenoble-Alpes, Grenoble, France; 2 Service d’épidémiologie et évaluation médicale, Centre Hospitalier Universitaire Grenoble-Alpes, Pavillon Taillefer, La Tronche, 38700 France; 3 Service des maladies infectieuses et tropicales, Centre Hospitalier Universitaire Grenoble-Alpes, Pavillon Taillefer, La Tronche, France; 4 Service de maladies infectieuses, Centre Hospitalier d’Annecy, Annecy, France; 5 Service des maladies infectieuses et tropicales, Centre Hospitalier Métropole Savoie, Chambéry, France; 6 Service de maladies infectieuses, Centre Hospitalier Universitaire de Saint-Etienne, Saint- Etienne, France; 7 Maladies Infectieuses et Réanimation Médicale, Hôpital Pontchaillou, Centre Hospitalo-Universitaire, Rennes, France; 8 Maladies Infectieuses, Centre Hospitalier Universitaire de Nice, Nice, France; 9 Médecine Interne Infectieuse et Tropicale, MiiT médical selarl, Lyon-Villeurbanne, France; 10 Equipe Mobile d’Infectiologie, APHP, Hôpital Cochin, Paris, F-75014 France; 11 Service de Maladies infectieuses, Groupe Hospitalier Sud Ile de France, Melun, France; 12 Maladies infectieuses et tropicales, Centre Hospitalier d’Ajaccio, Ajaccio, France; 13 Laboratoire TIMC-IMAG, Université de Grenoble Alpes, Grenoble, France

**Keywords:** Infectious diseases, Hotlines, General practitioner, Antibacterial drug resistance, Intersectoral collaborations

## Abstract

**Background:**

Telephone hotlines in infectious diseases (ID) are part of antimicrobial stewardship programs designed to provide support and expertise in ID and to control antibiotic resistance. The aim of the study was to characterize the activity of the ID hotlines and estimate their usefulness for general practitioners (GPs).

**Methods:**

This was a multicenter prospective observational study in different French regions. ID teams involved in antimicrobial stewardship with a hotline for GPs were asked to record their advice from April 2019 to June 2022. In these regions, all GPs were informed of the ID hotline’s operating procedures. The main outcome was usage rate of the hotlines by GPs.

**Results:**

Ten volunteer ID teams collected 4138 requests for advice from 2171 GPs. The proportion of GPs using the hotline varied pronouncedly by region, from 54% in the Isere department, to less than 1% in departments with the lowest usage. These differences were associated with the number of physicians in ID teams and with the age of the hotline. These results highlighted the value of working time as a means of ensuring the permanence of expertise. The main reasons for calling were: a diagnostic question (44%); choice of antibiotic (31%). The ID specialist provided advice on antibiotic therapy (43%) or a proposal for specialized consultation or hospitalization (11%).

**Conclusions:**

ID hotlines could help to strengthen cooperation between primary care and hospital medicine. However, the deployment and perpetuation of this activity require reflection concerning its institutional and financial support.

## Background

Antibiotic resistance, a natural phenomenon accelerated by the use of antibiotics, is a global public health issue. Worldwide, approximately 1.3 million deaths each year are estimated to be related to infections with antibiotic-resistant bacteria [1].

In order to control and prevent antibiotic resistance, international organizations such as the World Health Organization and the European Council of Disease Control recommend implementation of nationwide antimicrobial stewardship programs [2]. The concept of “*antimicrobial stewardship*” was introduced in the 1970s, and refers to programs designed to control bacterial resistance and improve antibiotic use [3].

Antimicrobial stewardship is an educational approach that includes audit and feedback actions, continuing education, infectious disease (ID) counseling and the use of information technology tools [4, 5]. In a systematic review of the literature, Davey et al. [4] showed that hospital-based antimicrobial stewardship programs decreased antibiotic prescription duration and average length of hospitalization, and that these interventions did not increase mortality.

In France, a law enacted in 2013 [6] requires designation in each hospital of a referent in antibiotic therapy, a physician or a pharmacist. His or her tasks are to promote appropriate antibiotic use, prescriber advice and medical staff training, as well as antibiotic consumption and bacterial resistance monitoring within the hospital. In this context, ID specialists have developed teleconsulting activities to guide prescribers in antibiotic treatment or antibioprophylaxis [7]. At the hospital, appropriate follow-up of ID specialists’ recommendations decreases consumption of anti-infectives [8, 9] and improves the overall quality of patient management [10, 11].

The problem of antibiotic resistance likewise arises in ambulatory medicine. In 2018, France was the fourth most antibiotic-consuming country in Europe, and 80% of prescriptions came from primary care [12]. Several studies have shown that antibiotic prescriptions in general practice were not optimal [13, 14]. For example, analysis of indicators of the appropriateness of antibiotic prescriptions by French GPs showed wide variations, from 9 to 75% of acceptable antibiotic therapy, depending on the situation and the GP [15]. The new French National Strategy 2022–2025 [16] for the prevention of antibiotic resistance includes the deployment of regional antibiotic therapy centers (CRATBs) specifically designed to improve antibiotic use in general practice.

The development of ID counselling for GPs is recommended as a means of combating antibiotic resistance in primary care medicine [12].

In 2000, the ID team of the Grenoble University Hospital set up a telephone hotline to respond to non-infectious disease physicians. This hotline was used more by GPs than by hospital physicians [17–19]. In this context, GPs expressed a need for support and expertise in infectiology [20]. GPs were generally satisfied with the system (97.9%), as were infectious disease specialists (94.7%) [20].

ID hotlines for GPs were gradually deployed in other French hospitals and regions. Deployment was based on the hypothesis that ID hotlines respond to a need of GPs and could contribute to improved antibiotic use.

In order to estimate the utility of ID hotlines for GPs, a French study group on antimicrobial stewardship programs proposed to ID teams to use a single dedicated computer database and to record the advice requested on the hotlines from 2019 to 2022.

The aim of the study was to characterize the activity of the ID hotlines, by analyzing the requests from GPs and the responses from ID specialists, in regions where they had volunteered to record hotline advice.

## Methods

### Study model

This was a multicenter prospective observational study based on the recording of GPs’ advice requests to an ID hotline.

### Intervention

ID teams involved in antimicrobial stewardship programs with a hotline for GPs were asked to record advice during the study period.

In the participating hospitals, the hotline existed before the survey. GPs could reach it via a single telephone number, usually the same as the in-hospital advice number.

The study was originally designed to last 1 year, but because of the Covid-related pandemic, it was extended to approximately 3 years, from April 2019 to June 2022.

We asked each volunteer ID team to describe the local organization of the ID hotline, particularly as regards opening hours. In each French department (French administrative subdivision) covered by a hotline, we identified all GPs working in ambulatory care through the French Health Insurance registry. A letter was sent to all GPs informing them of the hotline’s operating procedures and the call number. GPs were invited to use the hotline according to their needs. They were also informed that a research project was proceeding and that they could refuse to participate. ID specialists receiving a GP’s request for advice could respond with telephone advice or organize a consultation or hospitalization.

### Population

The study population consisted of all GPs who were invited, in April 2019, to use the hotlines to get ID advice. The analysis was based on the GP requests recorded by the ID teams from April 2019 to June 2022.

Advice given in response to calls from non-GP specialists or practitioners in health care facilities were not included in the study.

### Data collection

All requests to ID specialists made by GPs were recorded on a paper form, then entered into the AIRBUS database by a clinical research assistant. The AIRBUS database was a computer application developed on the Voozanoo platform (Epiconcept) accessible via the Internet and authorized to host medical data. It was also available on tablets or smartphones.

Data recorded were: characteristics of the caller; object of the call; date and time of the call; characteristics of the patient; characteristics of the advice given; recommendations proposed (hospitalization, consultation, advice on antibiotic therapy, diagnostic assistance, etc.); time spent giving the advice.

At the end of the registration period, a telephone interview was conducted by an investigator using a questionnaire with all the participating ID teams to collect information on the organization of the hotline, the difficulties encountered, and their perception of the usefulness of the hotline.

### Outcomes

The primary outcome is the usage rate, which is the proportion of GPs among invited GPs using the ID hotline at least once, in each study area.

Secondary outcomes were description of the objects of the call, recommendations given, and organization of the hotline in each ID team.

### Statistical analysis

The statistical unit was the advice given by the ID specialist. Qualitative variables were described by proportions with 95% confidence intervals; quantitative variables by median and 25th − 75th percentiles. Correlations between the usage rate and hotline characteristics were analyzed by Spearman’s test. The threshold of statistical significance was set at 5%, in a two-sided situation. Analyses were performed with Stata SE software (version 15.0, StataCorp, College Station, TX, USA).

### Ethics

The project received approval (on 24/09/2018) from the Ethics and Research Committee on Infectious and Tropical Diseases (IRB n° 00011642). The Voozanoo platform that supports the AIRBUS database was validated by the Commission Nationale Informatique et Liberté (CNIL). GPs could refuse the recording of their requests. Data confidentiality was ensured throughout the study.

## Results

### Study population

In France, among the fourteen ID teams that deployed an ID hotline, ten teams agreed to participate in the study, covering 10 French departments: Alpes-Maritimes, Corsica, Haute-Savoie, Ille-et-Vilaine, Isère, Loire, Paris, Rhône, Savoie, Seine-et-Marne.

All in all,13,216 GPs were invited to use the ID hotlines. During the study period, the participating teams received 4138 advice requests from 2171 GPs (Table 1). Out of these requests, 44% came from Isère, 22% from Haute-Savoie, 10% from Savoie and Loire, and 8% from Bretagne and Alpes-Maritimes. The other departments recorded less than 1% of the requests.

Apart from the Isère site, whose hotline was accessible 24h a day, 7 days a week, the other sites limited their availability to weekdays and working hours, with variations from one site to another.

**Table 1:** Distribution of calls and calling physicians

	Recorded calls		General Practitioners		Usage rate	Hotline opening days and hours	
			Guests (A)	Callers (B)	(B)/(A)		
Region (city)	n	%	n	n	%	Days	Hours
Isère (Grenoble)	1821	44	1582	852	**54**	7 days/week	24h/24
Haute-Savoie (Annecy)	909	22	909	348	**38**	Monday-Friday	9:00 am − 6:00 pm
Savoie (Chambéry)	420	10	1286	271	**21**	Monday-Friday Saturday-Sunday	9:00 am − 7:00 pm 9:00 am − 1:00 pm
Loire (St Etienne)	396	10	896	255	**28**	Monday-Friday	2:00 pm − 5:00 pm
Ille-et-Vilaine (Rennes)	332	8	600	243	**41**	Monday-Friday Saturday	9:00 am − 6:30 pm 9:00 am − 12:30 pm
Alpes-Maritimes (Nice)	206	8	1605	152	**9**	Monday-Friday	24h/24
Rhône (Lyon)	26	1	2441	24	**1**	Monday-Friday	8:30 am − 6:30 pm
Paris	19	< 0.5	3897	17	**0.5**	Monday-Friday	9:00 am − 6:30 pm
Seine-et-Marne (Melun)	7	< 0.5	1109	7	**1**	Monday-Friday	9:00 am − 7:00 pm
Corsica (Ajaccio)	2	< 0.5	364	2	**1**	Monday-Friday	9:00 am − 6:00 pm

### Usage rate of the hotline

During the study period, among 13,216 invited GPs, 2171 GPs used the ID hotline (16,4%). Usage rate varied between centers. More than half of the GPs in Isère used the hotline (54%). The usage rate was lower in the other departments, 38% of the GPs in Haute-Savoie, 41% in Ille-et-Vilaine, 28% in the Loire and 21% in Savoie. GPs in the other remaining departments made very little use of the hotline.

Among the 2171 GPs having called the hotline at least once, 63% were women. The number of calls by a single physician varied from 1 to 15: 65% made 1 call, 16% − 2 calls, 8% − 3 calls, 4% − 4 calls, 4% − 5 or 6 calls, the others (3%) made between 7 and 15 calls.

### Characteristics of the requests

**Fig. 1:**
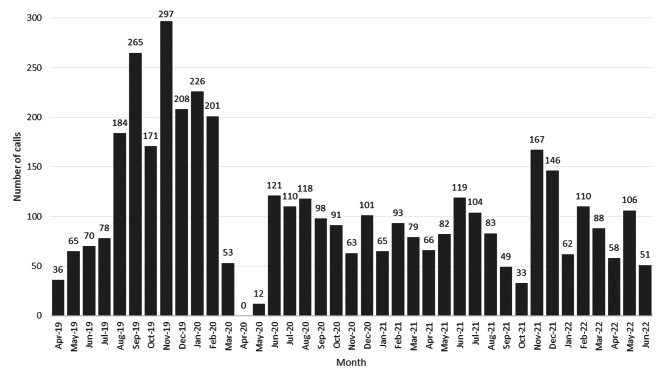
Monthly distribution of calls during the study period

The monthly number of recorded calls varied throughout the study (Fig. 1). The hotline was most widely used between August 2019 and February 2020, with a peak in November 2019. Recordings then dropped from March to May 2020 – the period of COVID lockdown – and subsequently increased to between 50 and 120 calls per month.

During the week, calls were mainly made from Monday to Friday and were evenly distributed over the days of the week (between 18% and 23%, with the highest rate on Monday). During the day, calls were mainly from 10:00 am to 12:00 pm and from 2:00 pm to 4:00 pm (26%), then from 4:00 pm to 6:00 pm (22%).

Patients for whom GPs sought advice were 51% female, with a median age of 50 years (IQR [31; 69]), 5% of them with known multidrug-resistant bacteria. The main reasons for calling were a diagnostic question (44%), choice of antibiotic (31%), vaccination (14%), general information (11%), organizing a consultation (6%), hospitalization (3%) and, finally, management of a blood exposure accident (2%).

### Response characteristics

During the same call, the ID specialist could provide several answers (Table 2). In the majority of cases (43%), the response included advice on antibiotic therapy (do not treat, initiate, stop, continue or optimize treatment). In 11% of cases, the response included a management proposal; either a consultation on infectious diseases (7%) or a consultation in another specialty (2%), or hospitalization in an ID unit (2%) or in another specialty (1%). For the other calls, ID physicians helped to diagnose (28%) and provided various types of advice (17%), mainly concerning vaccination, Covid or the adaptation of non-antibiotic treatments. ID physicians spent a median of 6 min per call (IQR [5; 10] minutes) on the telephone.

**Table 2:** Infectious disease (ID) specialist responses (n = 5239 responses; one call may result in multiple suggestions)

Responses (n = 5239)	n	%
**Advice on antibiotic therapy**	**2270**	**43%**
No treatment required	744	14%
Start antibiotic therapy	723	14%
Optimization of the treatment	487	9%
*Choice of another antibiotic*	*380*	*7%*
*Duration*	*212*	*4%*
*Dose*	*172*	*3%*
*De-escalation*	*11*	*< 0.5%*
*Oral relay*	*8*	*< 0.5%*
No change in current treatment	237	5%
Stop treatment	79	2%
**Proposal of care**	**598**	**11%**
Organization of an infectious disease consultation	356	7%
Hospitalization in infectiology	86	2%
Organization of a non-ID consultation	84	2%
Hospitalization other than in ID unit	72	1%
**Diagnostic help**	**1464**	**28%**
Proposal of complementary examinations	778	15%
Proposal of a diagnosis	668	13%
Interpretation of biological tests	18	< 0.5%
**Various suggestions**	**907**	**17%**
Vaccination	548	10%
COVID	107	2%
Adaptation of non-antibiotic treatment	98	2%
Other advice	154	3%

The diagnoses or clinical situations selected by the ID specialists were very diverse, headed by urinary tract infections (16%), skin infections (12%), Covid-19 and its vaccines (11%), fever and inflammatory syndromes (9%), digestive (8%) and pulmonary (8%) infections (Table 3).

**Table 3:** Clinical situations or diagnoses retained by the infectious disease specialist

Diagnoses given in 3812 calls*.	n	%
Urinary tract infection	601	16%
Skin infection	445	12%
Covid-19 and Covid-19 vaccine	409	11%
Fever and inflammatory syndrome	328	9%
Digestive infection	310	8%
Lung infection	304	8%
Bone and joint infections	178	5%
STDs** and gynecological infections	175	5%
Return from trip	172	5%
Vaccines	163	4%
Lyme and tick bites	140	4%
ORL infection	92	2%
Bites and other contagions	90	2%
Blood exposure accident	71	2%
Interpretation of a biological examination	63	2%
Non-infectious problem	57	2%
Other viral infections (EBV, CMV, HSV, VZV, flu)	55	1%
Parasitic infection	46	1%
Hepatitis	44	1%
Adenopathy	34	1%
Tuberculosis	30	1%
Nosocomial infection	26	1%
HIV	23	1%
Focal infection (abscess, endocarditis, meningitis, etc.)	13	< 0,5%
Other	149	4%

Missing data: * n = 326 (8%)

** STDs = Sexually Transmitted Diseases

### Factors associated with usage rate

**Fig. 2:**
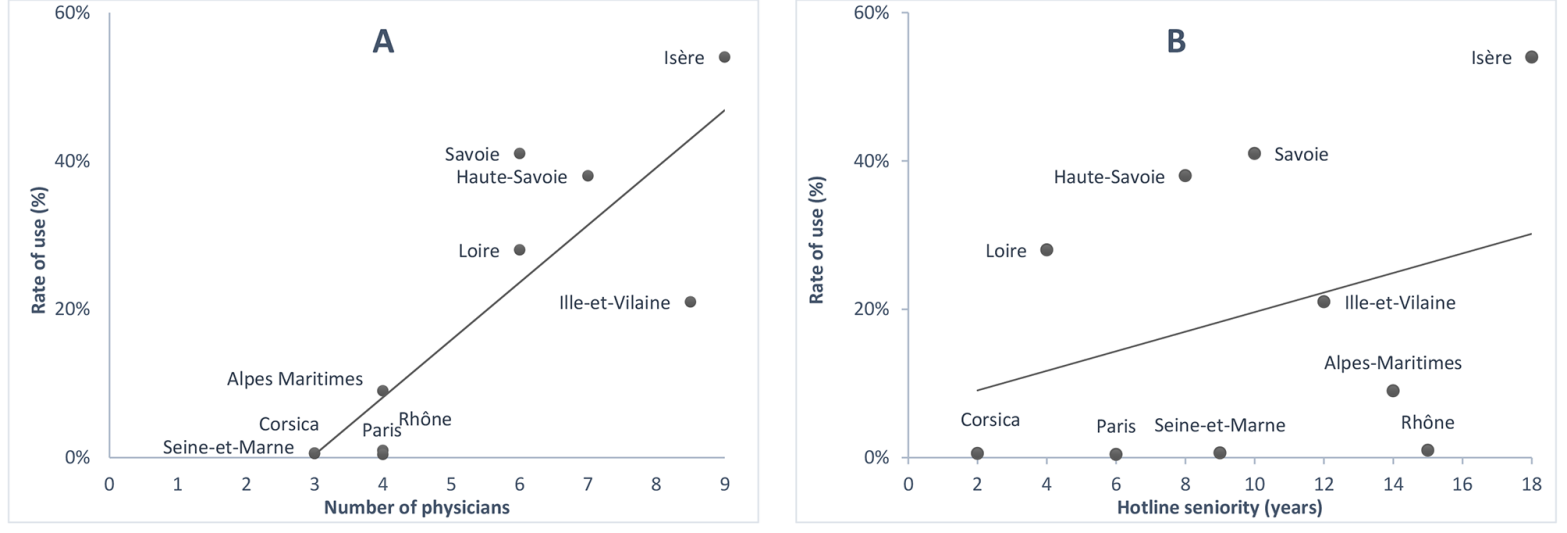
Usage rate of the hotline by general practitioners: **(A)** according to the number of infectious disease physicians on the team (r = 0.92; p < 0.001); **(B)** according to the age of the hotline (r = 0.47; p = 0.17)

Usage rate was correlated with the number of ID physicians on the hotline ID team, ranging from 9 to 2 (*r =* 0,92; ρ < 0.01) (Fig. 2). Usage rate also increased with hotline age, which ranged from 2 to 18 years, but the correlation was not significant (*r* = 0.47; p = 0,17). Hotline availability varied from 15 to 168 hours a week depending on the center. Correlation of this availability with usage rate was positive (r = 0.39) but not significant (p = 0.27). The proportion of responses by a senior physician (versus a resident supervised by a senior), ranged from 14 to 100% and was not significantly correlated with usage rate.

In the survey conducted with all ten participating ID teams, following the end of the study period, each team reported difficulties recording the advice. According to their declarations during the interviews, lack of recorded advice could vary from 10 to 70% of calls, depending on the team. The main reasons were lack of medical time and the complexity of the written procedures for recording the advice. They did not necessarily have the opportunity to write it down straight away, leading to oversights. These difficulties were exacerbated by the Covid-19 crisis and the population containment instituted during lockdowns.

In some centers, the hospital or health authorities funded medical time to support the antimicrobial stewardship policy. Unfortunately, this support never covered the totality needs, particularly regarding responses to GP requests. Some teams did not receive any support and the giving of advice was added on to their other tasks. There is no specific funding dedicated to advice for GPs.

When asked about the educational value of hotlines, most ID teams felt that the hotline allowed GPs to resolve complex clinical cases and, above all, to maintain a city-hospital cooperation, but they did not necessarily believe in the value of the hotline as a means of improving antibiotic use. According to their statements, GPs do not call for simple clinical cases (classic urinary tract infections or upper/lower respiratory tract infections), which are, they said, the main areas of antibiotic misuse in general practice.

## Discussion

This study showed that only 16.4% GPs used the ID hotline. It also showed great variability in usage rate of ID hotlines by GPs, estimated by the number of counsels recorded in each department. While 54% of GPs in the Isère department used the hotline, less than 1% did so in other departments; the level of implementation of telephone consultation for GPs markedly differed from site to site. This result weakens the study. Usage rate only counts calls that were made by GPs and recorded by the ID team. Usage rate per department varies with two parameters: the number of calls and the capacity of the team to record them. Concerning the capacity of ID teams to record advice, the qualitative survey showed it to be a failure.

The main factor affecting hotline usage rate was the number of physicians in the ID team having given advice. According to ID teams, activities related to antimicrobial stewardship, including telephone consultation, represent additional work with little or no remuneration. In most Western countries, specialists working in hospitals are paid on the basis of inpatient care or formal consultations, whereas informal consultations are not [20–22]. While some teams have obtained the creation of medical positions to support antimicrobial stewardship programs in the hospital, this type of reinforcement does not involve activities with GPs. The absence of medical reinforcement and financial support also impacts the possibility of tracing hotline activity. As the recording of advice takes extra time, overworked teams, especially during the Covid-19 epidemic, have difficulty finding the time to do it. However, in the perspective of specific financing for this activity, it matters that advice be traced. In France, the health insurance system is currently setting up a system rating the advice given by specialists [23]. While remuneration concerns exchanges via secure messaging between GP and specialist, it does not concern telephone advice. Traceability seems all the more justified as informal consultations may reveal concerns not only about the reliability of recommendations, but also about the responsibility of the physicians providing medical advice [24].

Experiences with hotlines for GPs have been reported in the literature in specialties such as otolaryngology [25], pediatrics [24] and psychiatry [26]. Zanaboni [27] described the Telemaco project in Italy concerning access to specialists in rural areas. In France, we can cite the example of geriatric hotlines. They were introduced by a 2009 law designed to deal with an aging population and a growing demand for unscheduled care [28]. These hotlines strengthen city-hospital cooperation by providing access to direct hospitalization for GPs in case of acute pathology [29] and help to reduce emergency department (ED) visits for geriatric patients [30].

These studies have shown that specialized hotlines are effective in improving care and reducing healthcare costs, and that they are fundamental in facilitating access to specialists and in reducing ED admissions and hospitalizations [11].

Moreover, the above studies emphasized the importance of financial support to ensure the long-term hotline sustainability of. In France, state funding provides for the opening of geriatrician positions in mobile geriatric teams [29]. Some authors emphasize the need to value not only specialists’ work, but also GPs’ [24, 27].

Hotline usage by GPs seemed to increase with the age of the hotline. This raises the question of the usual difficulties in implementing organizational innovation. Time and resources are necessary for actors to take ownership of an innovation [31, 32].

Once reason that may explain this inertia is the need for long-standing ID teams to introduce this new activity in their organization. Although ID teams are used to providing informal hospital-based consultations, setting up a hotline involves specific organization and consumes medical time. The age of the device also affected GPs’ knowledge of the system. Doctors who know the relevant phone numbers and have previously used the ID hotline have better integrated it into their practices.

The deployment of ID hotlines is part of a national policy aimed preventing antibiotic resistance. One of the axes of the 2022–2025 strategy concerns the proper use of antibiotics in general practice [16]. It includes online prescription assistance tools and the establishment of CRATBs dedicated to training, information and advice on antibiotic therapy for GPs. To the best of our knowledge, this is the first prospective multicenter study concerning the deployment of ID hotlines to primary care medicine. As an exploratory study, it provides information on GPs’ needs for ID advice and on the capacities of ID teams to record calls.

The main study limit is the under-recording of given advice, leading to underestimation of hotline activity. This was associated with the additional work time needed to record advice and was exacerbated by the Covid-19 epidemic, which mobilized the ID teams and impacted their organization. Nevertheless, the recordings retained drew an accurate picture of hotline activity.

The second limit is related to missing data: some of the recorded advice remained incomplete, which may have led to uncertain estimation.

Some ID teams with an ID hotline did not wish to participate in the study, which is likely to have led to selection bias.

Finally, the external validity of the results is questionable. Although the study was multicentric, it took place in a single country; the data and reflections are perhaps specific to the organization of the French healthcare system.

## Conclusions

The implementation of ID hotlines is part of antimicrobial stewardship programs in hospitals. Extension of these hotlines to primary care medicine is supported by the health authorities and could help to strengthen cooperation between ambulatory and hospital medicine. However, the authors found that without dedicated resources, implementation was difficult. Enlistment of economic support – by creating medical jobs to reinforce the ID teams and/or by remunerating the advice – seems to be a prerequisite for sustainable development of the ID hotline.

## Data Availability

Dataset generated and/or analyzed during the current study are not publicly available due to GPs’ identifying data but are available from the corresponding author on reasonable request.

## List of abbreviations

ID: Infectious Diseases
GPs: General practitioners
